# Tissue Resident CD8 Memory T Cell Responses in Cancer and Autoimmunity

**DOI:** 10.3389/fimmu.2018.02810

**Published:** 2018-11-29

**Authors:** Aleksey Molodtsov, Mary Jo Turk

**Affiliations:** Department of Microbiology and Immunology, The Norris Cotton Cancer Center, Geisel School of Medicine at Dartmouth, Lebanon, NH, United States

**Keywords:** T_RM_, TIL, CD103, T_CM_, melanoma, vitiligo, immunotherapy, biomarker

## Abstract

Resident memory (T_RM_) cells are a distinct tissue-localized T cell lineage that is crucial for protective immunity in peripheral tissues. While a great deal of effort has focused on defining their role in immunity to infections, studies now reveal T_RM_ cells as a vital component of the host immune response to cancer. Characterized by cell-surface molecules including CD103, CD69, and CD49a, T_RM_-like tumor-infiltrating lymphocytes (TILs) can be found in a wide range of human cancers, where they portend improved prognosis. Recent studies in mouse tumor models have shown that T_RM_ cells are induced by cancer vaccines delivered in peripheral tissue sites, or by the depletion of regulatory T cells. Such tumor-specific T_RM_ cells are recognized as both necessary and sufficient for long-lived protection against tumors in peripheral tissue locations. T_RM_ responses against tumor/self-antigens can concurrently result in the development of pathogenic T_RM_ responses to self, with a growing number of autoimmune diseases and inflammatory pathologies being attributed to T_RM_ responses. This review will recount the path to discovering the importance of resident memory CD8 T cells as they pertain to cancer immunity. In addition to highlighting key studies that directly implicate T_RM_ cells in anti-tumor immunity, we will highlight earlier work that implicitly suggested their importance. Informed by studies in infectious disease models, and instructed by a clear role for T_RM_ cells in autoimmunity, we will discuss strategies for therapeutically promoting T_RM_ responses in settings where they don't naturally occur.

## Introduction

Cancer can be considered a disease of immune dysfunction, with a failure of immune recognition leading to the outgrowth of malignant cells as tumors (1). Tumor development has been said to occur in three distinct steps: inefficient elimination of early transformed cells, development of a state of equilibrium between tumor cells and immune cells, and tumor escape from immune pressure (2). While innate immune cells are important for early tumor immune surveillance, T cells are fundamentally recognized for their crucial role in the antigen-specific recognition and elimination of malignantly transformed cells (2). Indeed a wealth of studies from humans and mouse models establishes a particularly potent role for CD8 T cells in controlling the outgrowth of malignancies (3).

The recent success of T cell immune checkpoint inhibitor (ICI) therapies for cancer has revealed CD8 T cells as potent mediators of immunity against advanced cancers (4, 5). Following effective priming in lymph nodes, T cells traffic to tumors and other peripheral tissues. In a growing number of cases, CD8 T cells have been shown to mediate the regression of large bulky tumors, resulting in durable long-term disease remissions (5). The persistence of such responses is fundamentally thought to be based on the ability of T cells to act as potent effectors and, subsequently, generate long-lived memory (6). T cell memory is antigen-specific, and can provide durable host-wide protection. As the field of cancer immunotherapy advances rapidly, it is now crucial to understand how the dissemination and maintenance of tumor-specific T cells can be optimally achieved.

Studies in infectious disease models have provided a wealth of information regarding memory CD8 T cell generation and localization. Classical definitions of memory T cells derive from the belief that T cells localize and recirculate predominantly throughout the blood and secondary lymphoid organs (7, 8). Such memory T cells were traditionally defined as being comprised of both central memory (T_CM_) and effector memory (T_EM_) subsets (9). T_CM_ cells were shown to persist and recirculate through the blood, bone marrow, lymph nodes, and spleen; whereas T_EM_ cells were shown to recirculate predominantly through blood, and peripheral tissues (10). In the early 2000's, large and persistent populations of antigen (Ag)-specific CD8 T cells in peripheral tissues were initially classified as T_EM_ cells in recirculation from the blood (8, 11).

These early classifications of T cell memory were quickly brought to bear on the question of what T cell subset provides the best immunity against cancer. A series of mouse adoptive T cell therapy studies published a decade ago showed that *in vitro* activated melanoma Ag (gp100)-specific T_CM_-like CD8 cells have a greater ability to control established melanomas in comparison with clonally-identical T_eff_/T_EM_-like cells (12, 13). Subsequent work in humans identified a third major subset of memory T cells known as stem cell-like memory (T_SCM_) cells (14). This less-differentiated T cell subset was capable of generating both T_CM_ and T_EM_ cells, and was shown in adoptive immunotherapy studies to have even greater anti-melanoma potency as compared with T_CM_ cells (15, 16). However, these early studies relied on definitions of memory that had been generated from a myopic focus on blood and lymphoid tissues. The concept that tumor-specific T cells could persist in peripheral tissues and tumors, without recirculation from the blood, was not yet being seriously considered.

Studies in viral models have now revealed a distinct lineage of memory T cells that resides in peripheral tissues and can provide orders of magnitude stronger protection than their T_CM_ cell counterparts (17). It is now recognized that peripheral host cells are surveyed overwhelmingly by T_RM_ cells that vastly outnumber their recirculating counterparts in peripheral tissues (18). The role of these tissue-resident memory (T_RM_) cells in immune responses against cancer is only beginning to be explored. However, early studies have revealed that T_RM_ cells are induced by vaccination, present in human tumors, and sustained by the same molecular mechanisms that were defined by infectious disease models. As the concepts of tumor immunity and autoimmunity remain closely linked, a better understanding of T_RM_ responses to cancer has also provided new insights regarding a role for T_RM_ cells in autoimmune disease. In turn, lessons regarding T_RM_ responses in autoimmune disease have begun to inform the field of tumor immunotherapy.

The goal of this review is to discuss new advances in our understanding of resident-memory T cells as they pertain to cancer immunity and associated autoimmunity. In addition to discussing recent studies that have directly implicated T_RM_ cells in anti-tumor immunity, we will highlight key early studies that implicitly suggested a contribution from T_RM_ cells before their existence was known. As the field has grown out of studies in infectious diseases, we will draw heavily on such models in forming the groundwork for studies in cancer. The focus of this article will be on CD8 T_RM_ cells as key mediators of the anti-tumor response, but not to imply an unimportant role for CD4 T cells. While CD4 T_RM_ cells have been described in multiple infectious disease settings (19), their role in immunity to cancer remains as yet undefined.

## Features of T_RM_ cells in infectious disease models

CD8 T_RM_ cells are defined based on their long-term persistence in peripheral tissues without recirculation from the blood. Since the earliest discovery of extra-lymphoid memory T cells in peripheral tissues of mice infected with vesicular stomatitus virus (VSV), and listeria monocytogenes (LM) infections (11), T_RM_ responses have been documented in response to a myriad of infections including lymphocytic choriomeningitis virus (LCMV) (20, 21), herpes simplex virus (HSV) (20, 22, 23), chlamydia (24), influenza (23, 25), vaccina virus (VACV) (17), human immunodeficiency virus (HIV) (26), tuberculosis (TB) (27), mouse cytolomegalovirus (MCMV) (28), and human papilloma virus (HPV) (29, 30). Thus, the formation of T_RM_ responses upon productive host infection can be viewed as a rule rather than an exception.

### Phenotypic features of T_RM_ cells

As a unique memory T cell lineage, CD8 T_RM_ cells can be distinguished from other T cell subsets based on their cell surface phenotype. Like all memory T cells, T_RM_ cells are differentiated from naïve T cells based on their expression of CD44; a marker of antigen experience (31). T_RM_ cells also lack expression of CD62L (L-selectin); which differentiates them from naïve T cells and T_CM_ cells that require CD62L for entry into secondary lymphoid organs (10). To distinguish T_RM_ cells from effector and T_EM_ cells, more detailed phenotypic considerations are necessary, and tissue retention markers; most notably CD103 and CD49a (VLA-1) are typically used. CD103 is a TGF-β induced molecule that promotes T_RM_ cell tissue retention by binding to e-cadherin, which is expressed on normal host epithelial cells (32). CD49a promotes tissue retention and survival through binding to collagenase type IV (33). While studies have largely focused on CD103 and CD49a as markers of tissue residency, it is important to note that their expression is not absolute, nor required. CD103 expression has been shown to be dispensable for T_RM_ formation in the liver (34) and gut (35), and CD49a is dispensable for T_RM_ formation in the lung (27). Additional retention markers have been used to identify T_RM_ cells, such as cutaneous leukocyte antigen (CLA) in the skin (36, 37) and LFA-1 in the liver (38). Thus, while T_RM_ cells are exclusively CD44^hi^ and CD62L^low^ they can express an array of tissue-specific retention markers, with no single marker being sufficient to definitively identify a T_RM_ population.

Another key phenotypic feature of T_RM_ cells is the expression of CD69, a marker of T cell activation, which blocks T cell expression S1PR1 (20). CD69 thus promotes tissue retention and residency by interfering with the ability to sense the S1P gradient that is essential for tissue egress (39, 40). Similarly, T_RM_ cells typically lack the expression of CCR7, which cooperates with S1PR1 for tissue egress through lymphatic vessels. However, as with CD103 and CD49a, CD69 can be dispensable for T_RM_ formation (18) and has been shown to be non-definitive in distinguishing recirculating T cells from T_RM_ cells in the steady state (41). In humans, a role for CD69 may be more pronounced than in mice, as peripheral tissue T_RM_ cells in healthy individuals overwhelmingly expressed (42).

T_RM_ cells occupy a unique niche in their tissue of residence, and take on a dendritic morphology that is uncharacteristic of circulating memory T cells (43). T_RM_ cells continually scan the peripheral tissue where an initial insult occurred (44), exhibiting limited migratory ability, and tending to accumulate at sites of antigen persistence (45). T_RM_ cells adapt well to their surroundings by exploiting the features of tissues in which they reside. In skin, T_RM_ cells have been shown to cluster around niches formed by keratinocytes, near hair follicles, which secrete IL-15, IL-7 (46), and TGF-β (47). In anatomical regions with a high tissue turnover rate, such as the lamina propria of the gut, immune cells such as macrophages support the formation of T_RM_ aggregates (48). In other barrier tissues, T_RM_ cells occupy *de novo* niches, such as repair-associated memory depots (RAMD) in the lung, and mucosa-associated lymphoid tissue (MALT) in the female reproductive tract (FRT) (49, 50). Localization to barrier sites of mucosal tissues exerts a metabolic burden that typically limits the persistence of T cells. However, T_RM_ cells utilize fatty acid beta-oxidative phosphorylation to support their longevity (51–53). In contrast to conventional memory T cells which conduct their own fatty acid synthesis, T_RM_ cells in the skin have been shown to express high levels of fatty acid binding proteins FABP4 and 5, to facilitate the necessary uptake of fatty acids (51). These properties of T_RM_ cells enable them to function in diverse peripheral tissue niches.

### Transcriptional profiles of T_RM_ cells

Transcriptional profiling has demonstrated that T_RM_ cells are distinct from their T_CM_ and T_EM_ counterparts, and thus represent a unique T cell lineage (20, 54). Although unique genes define T_RM_ subpopulations in different tissues (20), a core transcriptional signature has been proposed for T_RM_ cells (54, 55). This signature highlights the distinctive nature of T_RM_ cells as a hybrid between effector and memory cells, which are armed in an effector like-state even during quiescence (54, 55).

Master transcriptional regulators of T_RM_ cell differentiation across multiple tissue types include Hobit, Blimp1 (42), and Runx3 (54). In contrast, *Tbx21* (T-bet) and *Eomes*—the master regulators of effector and lymphoid memory T cell lineages—have been shown to impede the development of T_RM_ cells (56). Hobit and its homolog Blimp1 act in synergy as negative regulators of tissue egress, by directly binding to *S1pr1, Ccr7*, and *Tcf7* motifs in mice (55). Additionally, Blimp-1 has been shown to initiate cytotoxic function while Hobit maintains deployment-ready cytotoxicity in T_RM_ cells (57). On the other hand, Runx3 acts to promote the expression of T_RM_ tissue retention markers such as CD103 and CD69 (54). In addition to these canonical T_RM_ transcription factors, the NR4A family has also been shown to be highly upregulated in T_RM_ cells, with the absence of *Nr4a1* resulting in a reduced capacity to generate T_RM_ (58).

While most in-depth transcriptional analyses have, to date, been conducted in murine infectious disease models, transcriptional characteristics of T_RM_ cells from humans are also beginning to be reported. CD8 T_RM_ cells from human lungs showed high CD69 expression, and variable CD103 expression (59). These lung T_RM_ cells could be distinguished from their circulating counterparts by high levels of *GZMB, IFNG, TNF, and NOTCH1* transcripts, with NOTCH signaling shown to promote *IFNG* gene expression (59). Separate studies showed that CD69^+^ memory T cells across multiple tissues of human cadavers exhibit a conserved transcriptional profile including *ITGA1* (CD49a), *ITGAE* (CD103), and *PDCD1* (PD-1) expression (42). In contrast to mouse T_RM_ cells however, human cells lacked expression of *ZNF683* (HOBIT*)* and *PRDM1 (*BLIMP-1*)* (42). It is important to note that T_RM_ cell transcriptional signatures have been largely generated from pooled T cell samples, thus lacking single cell resolution and missing the complexity and heterogeneity that potentially exists within a T_RM_ cell pool. Single-cell RNA-sequencing of T_RM_ cells from mice and humans may, in the future, reveal heterogeneous CD8 T_RM_ cell subsets.

### Protective function of T_RM_ cells

T_RM_ cells have been shown to play a dominant role in protection against peripheral infections, in some cases mediating orders of magnitude stronger protection than lymphoid memory T cells (17). However, because infections generate both resident and circulating memory T cell compartments, specialized techniques have been needed to isolate the contribution of T_RM_ cells from that of their lymphoid memory counterparts (60). FTY720 is a small molecule S1PR antagonist that inhibits T cell egress from lymph nodes, and thereby prevents circulating memory T cell subsets from accessing peripheral tissues (61). In mice infected by VACV-OVA through skin scarification (s.s.), it was shown that treatment with FTY720 had no effect on protection against cutaneous viral re-challenge, indicating that skin T_RM_ cells are sufficient for long-lived protection (17). In the setting of influenza viral infection, a protective role for lung T_RM_ cells was also first established by studies involving FTY720 treatment (62). Low dose monoclonal antibody (mAb) depletion strategies can also be employed based on their ability to efficiently deplete circulating and lymphoid T cells, while sparing tissue-resident T cell niches (49). Using this technique, HSV skin infection was shown to generate long-lived protective immunity that was unperturbed by the depletion of circulating memory T cells (20). Lastly, the surgical joining procedure known as parabiosis, has been used to isolate the contribution of T_CM_ from that of T_RM_. Parabiosis allows the equilibration of circulating immune compartments between immune and naïve mice, thus transferring circulating memory to a naive recipient (8). Through elegant parabiosis studies in conjunction with the use of FTY720, skin CD8^+^ T_RM_ cells were shown to be superior to T_CM_ in protecting against cutaneous VACV re-infection (17). These fundamental studies established a crucial role for T_RM_ cells in mediating long-lived protection against peripheral infections.

## Resident memory T cell responses to cancer

**Figure 1:**
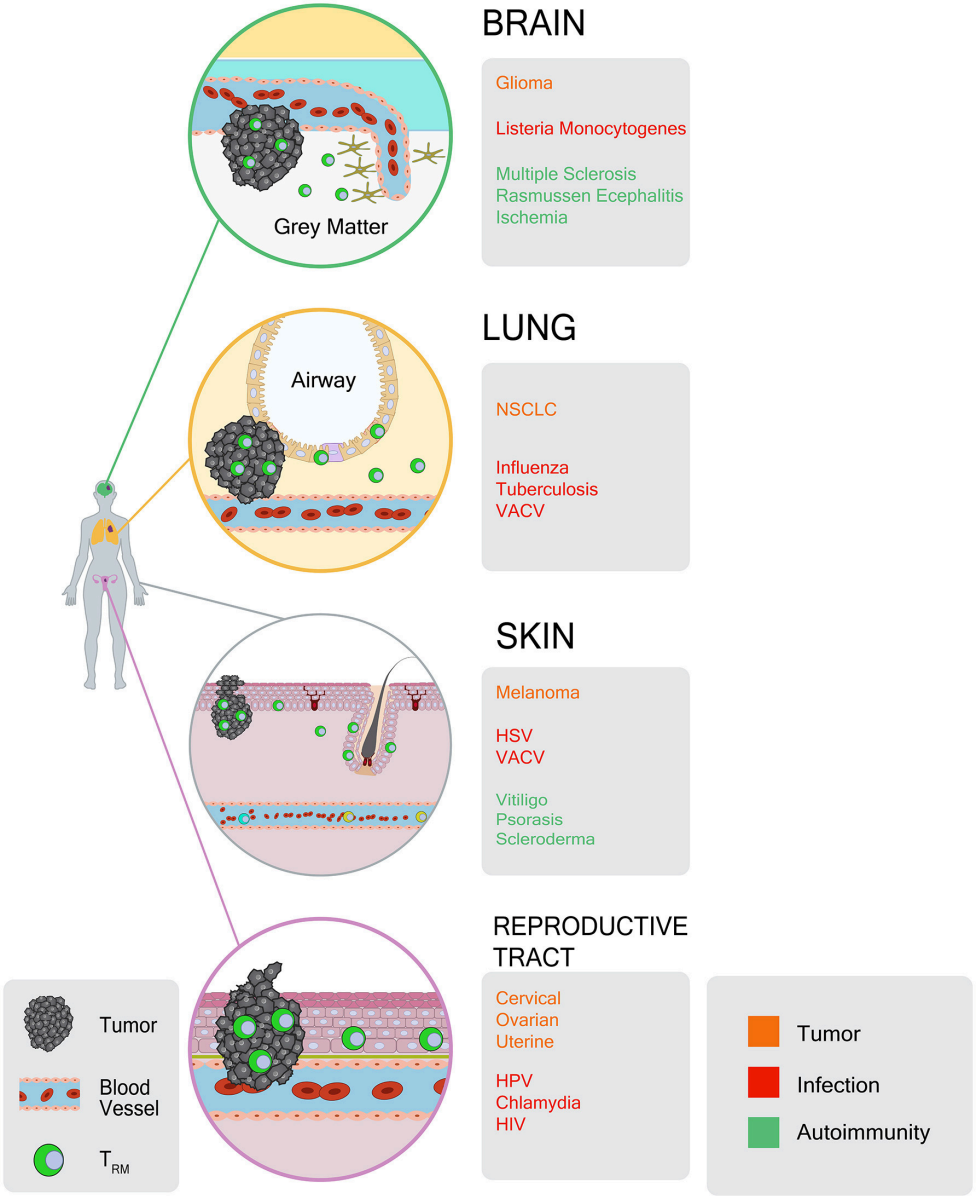
Atlas of T_RM_ responses in peripheral tissues and tumors. CD8^+^ T_RM_ cells occupy a diverse array of tissues and tumors. For each tissue location, gray boxes indicate: (1) tumor types in which T_RM_-like TILs have been identified (orange), (2) infections that give rise to T_RM_ responses (red), and (3) autoimmune or inflammatory conditions in which T_RM_ cells have been identified (green).

The above characteristics of T_RM_ cells have, more recently, been recognized for their relevance to cancer immunity. Indeed, as infections occur in peripheral tissues, so do cancers arise in the same tissues. As such, it stands to reason that populations of tumor-specific T_RM_ cells can occupy tumors themselves, and the tissues from which they arise (Figure 1). Despite this, the role of T_RM_ cells in mediating immunity to cancer has only recently been described.

Prior to the term “resident memory,” a growing body of literature had already identified CD103 expressing CD8 T cells within human tumors, and linked these cells to improved prognosis. With CD103 now recognized as a common T_RM_ cell marker, these studies can be viewed as the earliest evidence of tumor infiltrating lymphocytes (TILs) having T_RM_-like properties. Importantly, these findings formed the ground work for the extensive characterization of T_RM_ cell transcriptional signatures in a variety of human tumors, and mechanistic mouse work to establish a crucial role for this memory T cell subset in immunity to cancer.

### Identification of CD103^+^CD8^+^ TILs

Studies examining CD103 expression on tumor infiltrating lymphocytes date back 30 years, with an initial focus on the identification of cellular localization patterns within tumors. A 1988 analysis was the first to identify CD103-positive tumor infiltrating lymphocytes (TILs) (63). These studies found that a majority of TILs in gastrointestinal tumors were positive for CD103 (at the time referred to as HML1), and that these cells were localized throughout the tumor mass (63). A decade later, studies involving pancreatic cancer patients revealed that approximately 20% of all CD8 T cells in tumors expressed CD103 as well as CD45RO, a human memory T cell marker (64). Unlike the prior study (65) these studies showed CD103^+^ T cell aggregation in fibrous stromal tissue, and exclusion from tumor cell nests (64). Tumor-excluded T cell distribution was further supported by a 2001 analysis of bladder cancer samples in which the majority of CD103^+^CD8^+^ TILs were found on the periphery of the tumor, potentially suggesting immune failure (66). However, a 2003 study reported high levels of CD103^+^CD8^+^ T cells infiltrating intratumoral regions of microsatellite-unstable colorectal cancers (65). Later work revealed that CD103^+^ TILs were not limited to tumor masses, and could also be found in the ascites fluid of high-grade serous ovarian cancer (67). This early collection of studies established CD103 expression on CD8^+^ TILs in various solid cancers, but indicated their variable distribution throughout the tumor microenvironment (TME).

With CD103 substantiated as marker of tumor-infiltrating CD8 T cells, several early studies also sought to define the function of CD103 in the interaction between T cells and tumor cells. Attempts at defining these interactions focused predominantly on two molecules: TGF-β, as an inducer of CD103 expression (68, 69), and e-cadherin, as the only known binding partner for CD103 (70). An early study showed that TGF-β-induced CD103 expression significantly enhanced the lysis of e-cadherin-transduced pancreatic cancer cells by CD8 T cells *in vitro* (71). This was found to occur by increasing the adhesion of CTL's to tumor cells, a process which depended on the expression of both CD103 and e-cadherin (71). However, it remained unknown whether such interactions occur in response to tumor cell encounter, and whether Ag recognition plays a role in CD103 induction. Subsequent studies involving human HER2/NEU-specific T cell clones revealed that cognate antigen and TGF-β were both required for the *in vitro* upregulation of CD103 on T cells (72). Furthermore, a human colon cancer cell line was capable of secreting enough TGF-β to promote T cell expression of CD103 upon Ag-recognition *in vitro*, suggesting that tumor-derived TGF-β could induce CD103 expression on TILs (72). This study further showed that T cells exposed to TGF-β during priming, will re-express CD103 more readily upon recall (72). Follow-up studies in a human lung cancer xenograft mouse model showed that CD103 is induced upon T cell trafficking to tumors, and that *in vivo* neutralization of TGF-β impairs the recruitment of CD103^+^ CD8 T cells into the TME (73). These studies together substantiated the role of tumor-derived TGF-β in the induction of CD103 on intratumoral T cells.

While CD103 upregulation on TILs was shown to be TGF-β dependent, the mechanism governing this process in cancer still remained unclear. In 2014, it was shown that TGF-β induces Smad2/3 phosphorylation which allows nuclear translocation and binding to the proximal regulatory elements of CD103 (74). Indeed pSmad2/3 nuclearization was recently used as a marker of CD103^+^ TIL's in human cervical cancers, in which TGF-β signaling is abundant (75). The key role that TGF-β plays in CD103^+^ TIL biology was further validated using a tumor-specific human T cell clone, by showing that binding of TGF-β to its receptor promoted the recruitment and phosphorylation of integrin-linked kinase (ILK) to the CD103 intracellular domain, inducing integrin inside-out signaling that may further promote T_RM_ cell migration and function (76, 77).

Subsequent studies have also shed light on the putative role of CD103/e-cadherin interactions in promoting tumor cell killing. *In vitro* studies used CD103 expressing tumor-antigen specific T cell lines from lung cancer patients to demonstrate that e-cadherin expression was required by tumor cells for effective killing via granule polarization and exocytosis (78). It was separately shown that CD103 ligand engagement together with TCR binding, enhances the strength of human TIL/tumor cell interactions (79). CD103 engagement with e-cadherin was found to shape immunological synapse morphology, which was essential for the polarization of cytokine and lytic granules containing granzyme B and IFN-γ (79). A co-stimulatory role for CD103 was subsequently established by showing that CD103 triggering promotes phosphorylation of ERK1/2 kinases and phospholipase Cγ1 (PLCγ1)—a process that was required for cytotoxicity by tumor-specific human T cells (80). CD103-downstream signaling in TILs was separately shown to require paxillin phosphorylation and binding to the CD103 cytoplasmic tail, potentiating effector function against cognate lung tumor cells (81). Thus, ligation of CD103 has been proposed to enhance tumoricidal T cell functions *in vivo*.

While the above studies employed e-cadherin-expressing tumor cell lines and/or transfectants, it should be noted that the expression of e-cadherin is often lost on epithelial cells during malignant transformation and tumorigenesis (82). This may suggest that CD103-ecadherin interactions are unlikely to play a major role in the TME. This is underscored by analyses of bladder (66), ovarian (83), lung (84), and pancreatic cancer (85) specimens showing either a lack of co-localization between CD103-positivie TILs and e-cadherin expressing tumor cells, or a lack of correlation between e-cadherin expression levels and CD103^+^ TIL infiltration. However, some studies do report the contrary (86). Interestingly, CD103 has been shown to have unknown binding partners in peripheral tissue (87, 88), and one could speculate that such partners function in engaging CD103 on CD8 T cells in e-cadherin negative tumors, or negative regions within tumors. Alternately, following upregulation of CD103 by tumor-derived TGF-β, this integrin might serve as a marker of the T_RM_ transcriptional program, rather than a functional player that engages its ligand in the TME.

### Prognostic significance of CD103^+^CD8^+^ TILs

While early studies confirmed the existence of CD103^+^CD8^+^ TlLs in solid tumors, evidence that these cells had prognostic value for patients did not appear in the literature until 2014, when it was shown that this subset is strongly associated with survival in high-grade serous ovarian carcinoma (HGSOC) (83). In this study, CD103^+^ TILs were clearly localized to intratumoral regions, as opposed to associated tumor stroma. Interestingly, the presence of CD8^+^CD103^−^ cells conferred no benefit when compared to tumors devoid of all CD8 T cells, suggesting that the CD103^+^CD8^+^ subset dominates the protective response in HGSOC (83).

Studies followed in several tumor types, all demonstrating correlation between CD103^+^CD8^+^ cells localized in tumor nests (also referred to as “intraepithelial” regions), and improved patient prognosis. In urothelial (bladder) cancer, high intratumoral CD103^+^CD8^+^ TIL density was inversely correlated with tumor size and could be used to predict improved overall survival (86). Similarly, a large breast cancer study demonstrated that CD103^+^ TIL infiltration to intratumoral (but not stromal) regions of tumor masses was prognostic of survival in a basal-like subtype (89). Investigation of endometrial adenocarcinoma showed that CD103 expression clearly delineated CD8 T cells localized to intratumoral regions (as opposed to stromal regions), and was an independent predictor of improved survival, particularly for high risk disease (90). Intraepithelial CD103-positive TILs were separately showed to be a valuable biomarker for therapeutic response in cervical cancer patients undergoing chemotherapy and radiation therapy (91). In contrast to earlier studies, these studies all supported CD103 as a marker of T cells infiltrating tumor nests as opposed to stroma. Importantly, these studies highlighted the superior prognostic strength of CD103^+^CD8^+^ TILs as compared with total CD8^+^ TILs.

Several studies have also supported this association for lung cancer. Early work showed that total CD103^+^CD8^+^ TILs have prognostic value in non-small cell lung cancer (NSCLC) patients with regards to early disease-free survival (92). Further subsetting of NSCLC patients showed that high numbers of CD103^+^ TILs in tumor nests were an independent predictor of disease-free survival for patients with pulmonary squamous cell carcinoma (84). Interestingly, this study also reported a strong correlation between smoking status and increased density of CD103^+^ TILs (84). A separate study of human NSCLC tumors showed that CD8 T cells in intratumoral regions were more likely to express CD103 than those in stromal regions, which was again highly predictive of survival (93). Further validation of CD103 as a biomarker was provided through stratification of NSCLC patients in The Cancer Genome Atlas (TCGA) database, which showed that patients with high tumor *ITGAE* (CD103) expression have improved overall survival (93).

For pancreatic cancer, the association between CD103 expression and prognosis is less straight-forward. Whereas high numbers of total CD8 T cells predicted improved prognosis in pancreatic ductal cell adenocarcinoma (PDAC), CD103^+^CD8^+^ TIL numbers did not predict survival, nor did CD103^+^CD8^+^ TILs in intratumoral regions (85). Interestingly though, a high ratio of CD103^+^ TILs in intratumoral vs. stromal locations was predictive of prognosis, potentially indicating the importance of spatial relationships between T_RM_-like cell subsets in PDAC (85). Indeed T_RM_ cell localization to intratumoral regions would appear to situate them for optimal tumor control, although CD103^+^ TIL subsets may not be prognostic in all tumor types.

### Phenotypic characteristics of T_RM_-like TILs

More detailed phenotypic analyses of CD8^+^CD103^+^ TILs have revealed additional T_RM_-like characteristics of these cells. Investigation of HGSCOC, endometrial adenocarcinoma, and ovarian cancer, all showed that CD8^+^CD103^+^ TILs express high levels of the exhaustion marker PD-1 (75, 83, 90). CD8 T cells in pediatric glial tumors exhibited a CD45RO^+^ CD69^+^ CCR7^−^ T_RM_-like phenotype, in addition to multiple inhibitory checkpoints including PD-1, PD-L1, and TIGIT (94). Detailed flow cytometric and qPCR analyses of CD103^+^CD8^+^ TILs from lung cancer patients also revealed high levels of *PDCD1* (PD-1) and *HAVCR2* (TIM3), and low levels of the tissue egress marker *S1PR1* (92). Using an immunofluorescence technique to visualize T_RM_ in NSCLC, co-expression of CD49a (VLA-1) was identified on CD103^+^CD8^+^ T cells (93). This study also showed elevated levels of PD-1 and TIM-3 on CD103^+^CD8^+^ TILs as compared to CD103-negative TILs. Accordingly Cy-TOF analysis of melanoma-infiltrating T cells showed that a CD69^+^ subset (among which ~50% expressed CD103), co-expressed high levels of inhibitory checkpoint molecules CTLA-4 and PD-1 (95). Many of these studies have thus hypothesized that T_RM_-like TILs are key targets of immune checkpoint inhibitor therapy.

It is important to note that inhibitory checkpoint molecules, while overwhelmingly expressed on T_RM_-like TILs, have demonstrated somewhat more variable expression on T_RM_ cells in peripheral infection models. PD-1 is expressed on CD103^+^CD8^+^ brain T_RM_ cells in response to listeria monocytogenes, MCMV, and VSV infection (96, 97). Interestingly, upregulation of PD-1 on brain T_RM_ cells is independent of chronic antigen stimulation or inflammation (98). However, a study of VSV infection found PD-1 expression absent on T_RM_ cells in the brain (99). In the skin, HSV infection induces T_RM_ cells that express PD-1 (20, 100), and CTLA-4 (20). However, VACV infection produced PD-1-negative T_RM_ cells in the skin (17), and LCMV infection induced T_RM_ cells in the small intestine that lacked PD-1 (101). Thus, inhibitory checkpoint molecule expression does not appear to be a defining feature of T_RM_ cells in peripheral infection models. Moreover, it remains unclear if PD-1 expression levels differ between CD103^+^ cells in normal peripheral tissues and those in tumors.

Considering the widespread expression of inhibitory checkpoint molecules on CD103^+^ TILs, it has remained unclear if these cells represent true T_RM_ cells or, rather, exhausted TILs that simply express CD103 in the TGF-β rich TME. Indeed no studies as-yet have demonstrated the long-term persistence of CD103^+^ CD8 T cells in tumors, without ongoing input from circulation—the true hallmark of a T_RM_ response. In HGSCOC tumors it was further shown that CD103^+^ TILs have phenotypic characteristics of varying T cell differentiation states including T_CM_, T_EM_, and T_RM_ cells (75), which may suggest that these cells are replenished from circulation. In the next section we will highlight several studies that transcriptionally profiled CD8^+^ TILs to generate a more comprehensive profile of their gene expression signature. These results, although varied across different tumor types, collectively support the conclusion that subsets of TILs are regulated by T_RM_ transcriptional programs.

### Transcriptional profiles of T_RM_-like TILs

Transcriptional profiling of TILs from NSCLC tumors has provided new insights into the characteristics of T_RM_-like TILS that extend beyond lung cancer. NSCLC tumors with a high TIL infiltration score were shown to have more pronounced gene expression characteristics of T_RM_ cells including higher transcript levels for *ITGAE (CD103), CD69, ITGA1 (*CD49a*), CXCR6, PDCD1 (PD-1), HAVCR2* (TIM3), *LAG3*, and *TIGIT*, but lower expression of *KLRG1, CCR7, SELL (*CD62L), and *S1PR1* (102). Even among tumors with high CD8 T cell density, high expression of CD103 conferred patient survival advantage (102). Focusing on CD103^hi^ CD8 TILs, this study also identified elevated expression of components of the NOTCH signaling pathway, as well as CD39, the cell surface ectonucleotidase that dephosphorylates ATP (102). This CD103^+^CD39^+^ TIL subset was the focus of a subsequent study that identified this populations in head and neck squamous cell carcinoma (HNSCC), melanoma, HNSCC, ovarian, lung, and rectal cancer tumors (103). In HNSCC patients, the CD103^+^CD39^+^ TIL population was found to be a better predictor of survival as compared with CD39 and/or CD103-negative CD8 T cell subsets (103). Transcriptional analysis of this sorted CD103^+^CD39^+^ subset from five tumors (HNSCC and ovarian) revealed enrichment of gene transcripts associated with exhaustion and reduced expression of T cell recirculation associated genes, suggesting that CD103^+^CD39^+^ TILs may have the most pronounced T_RM_-like character in tumors (103).

TIL characterization through bulk RNA-sequencing provides a wealth of transcriptional data, but obstructs the detection of small heterogenous populations within the TME. On the other hand, single-cell RNA sequencing (scRNA-seq) allows for fine resolution of TIL sub-populations at a cost of failing to detect poorly expressed transcripts. Indeed scRNA-seq of over six thousand TILs isolated from two patients with triple negative breast cancer (TNBC) showed at least four differential clusters of CD8 T cells, all expressing CD69 (104). One TIL cluster, identified based on high CD103 expression, was shown to express low levels of transcripts for *KLRG1* and *SELL* (CD62L), as well as tissue egress related genes, *KLF2* and *S1PR1* (104). This TIL subset also expressed high transcript for inhibitory checkpoint genes *HAVCR2, PDCD1, CTLA4, TIGIT*, and *LAG3*, and cytotoxicity-related genes *GZMB* (granzyme) and *PRF1* (perforin). Coupled with bulk RNA-seq data on sorted TIL populations, this study showed that CD8^+^CD103^+^ TILs in breast cancer exhibit multiple features of T_RM_ differentiation. Importantly, this T_RM_-like TIL gene signature was predictive of survival in TNBC patients from the METABRIC consortium, and could be used to distinguish melanoma responders to ICI therapy (104).

Further highlighting the heterogenicity of the TIL population, another study sequenced >12,000 TILs from twelve NSCLC tumors and identified seven clusters with one expressing the T_RM_ specific transcription factor, *ZNF683* (*HOBIT*) (105). In contrast to the above study in TNBC, HOBIT-expressing lung T_RM_-like TILs expressed low levels of CD103 and showed reduced expression of PD-1 as compared to other CD8 clusters, suggesting a unique transcriptional program in NSCLC T_RM_-like TILs (105). As in TNBC, the scRNA-seq derived gene signature was used to stratify patients in a TCGA lung adenocarcinoma (LUAD) dataset, to show that enrichment of a CD8-ZNF683 gene-signature is predictive of survival when compared to other TIL derived signatures (105).

Taken together, the above studies highlight variability in T_RM_-like TIL gene signatures in cancer. Discrepancies likely arise due to unique tumor microenvironmental factors associated with individual patients, potentially relating to tumor tissue of origin, stage, and mutational status and/or burden. In comparing T_RM_-like TILs to bona fide T_RM_ cells in normal peripheral tissues, it is also important to note that tumors, by their nature, lack many of the structural and molecular features of normal tissues that provide a hospitable niche for T_RM_ cells. Thus, one might expect that T_RM_-like TILs would never perfectly match T_RM_ cells that reside in the normal tissue counterpart of a tumor. Despite this, the discovery that TILs with features of T_RM_ cells portend improved patient survival across multiple tumor types, underscores the importance of these cells, and represents a key recent advance in the field of cancer immunology.

### Role of T_RM_ cells in mediating immunity to cancer; lessons from mouse models

While the above studies provide strong correlative associations between T_RM_ cells and improved patient survival, until very recently, formal evidence that T_RM_ cells can mediate immunity to cancer was lacking. In retrospect, preclinical cancer vaccine studies published 15 years ago, inferred the crucial contribution of a long-lived, tissue-localized T cell population, however the importance of resident memory had not yet been recognized. This section will illustrate the path to our discovery of a role for T_RM_ cells in mediating durable anti-tumor immunity.

#### Early indications of T_RM_ cells from studies of cancer vaccination route

One of the earliest studies to infer T_RM_ cell responses to cancer, published in 2003, revealed that melanoma-specific memory CD8 T cells distribute to distinct tissue locations, depending on the route of vaccination (106). By administering a peptide-pulsed dendritic cell (DC) vaccine via various routes, it was shown that only subcutaneous vaccination could reliably protect against a subcutaneous melanoma tumor rechallenge (106). This concept of regionally localized tumor-specific CD8 T cell memory was revolutionary at the time, although lymphoid tissues remained the focus of this early work. As such, tumor protection in these mice was attributed to memory populations in local lymph nodes, rather than in peripheral tissues (106).

Subsequent work extended the concept of tissue-localized tumor immunity, while further demonstrating that tumor-specific CD8 T cells could localize to peripheral tissue. In a 2010 study, mice infected through various routes with recombinant vaccinia virus (rVACV) expressing OVA_257−264_, were challenged with an OVA-expressing B16 melanoma cell line 6 weeks later (107). Reminiscent of the 2003 study, optimal protection against dermal melanoma rechallenge was only afforded by prior infection in the skin (107). This study further identified OVA-specific T cells in the skin, and referred to them as “skin-resident T_EM_ cells” (107). While it was inferred that such T_EM_ cells could mediate tumor protection in the skin, no formal experiments were performed to isolate the effects of this tissue-resident population from those of the lymphoid memory T cell compartment, both of which were present in tumor-bearing mice.

The field again approached this concept in 2013, with studies showing that growth of orthotopic head and neck TC1 tumors (implanted in the tongue) could only be inhibited when prior vaccination had been delivered via the intranasal (i.n.) route (108). In this study, i.n. vaccination with Shiga toxin B subunit fused to the HPV16-E7 tumor/viral peptide (STxB-E7) gave rise to E7_39−47_-specific CD8^+^ T cells in mucosa-draining lymph nodes. These tumor-specific T cells expressed CD103 and CD49a however, in contrast to the 2010 study, T cell responses were not analyzed in peripheral (mucosal) tissue itself. Interestingly, mAb-mediated blockade of CD49a was shown to block CD8 T cell infiltration into TC1 tumors during acute STxB-E7 treatment, indicating CD49a as an important determinant of T cell infiltration into tumors (108). One other report in 2016 similarly showed that *in vivo* mAb-mediated blockade of CD49a or CD103 significantly impaired the control of subcutaneous B16-OVA tumors, supporting important roles for these molecules in T cell mediated anti-tumor immunity (109). While these studies revealed important new concepts regarding vaccine route and markers of tissue (or tumor) residence, the question of whether T_RM_ cells directly mediate tumor protection remained open.

#### A key role for T_RM_ cells in mediating anti-tumor immunity

Several notable studies in the past 2 years have now definitively demonstrated a role for T_RM_ cells in mediating immunity to cancer. Importantly, each of these studies employed techniques originally used in infectious disease models, to isolate the contribution of T_RM_ cells from that of the lymphoid memory compartment. In doing so, these studies illustrate a definitive role for T_RM_ cells in providing long-lived protection against multiple tumor types, and in various tissue locations.

In follow-up work to the 2014 study involving STxB-E7 vaccination, it was shown that intranasal vaccination indeed generates a large pool of Ag-specific memory T cells in lung mucosal tissue (93). This was in clear contrast to intramuscular vaccination, which generated effector-like T cells in the spleen. E7-specific CD8 T cells in the lung expressed T_RM_ markers including CD103 and CD49a, that were absent on T cells in the spleen. Further transcriptomic analysis of E7 Ag-specific CD8 T cells from the spleen showed that they expressed higher levels of lymphoid homing and tissue exit markers (i.e., *Sell* and *S1pr1*) compared with lung, while lacking adhesion and retention markers. Three key experiments were conducted to implicate T_RM_ cells in the recall response against E7-expressing TC1 head and neck tumors. First, FTY720 was used to illustrate a minimal contribution of circulating T cells to tumor protection (93). Second, *in vivo* mAb-mediated TGF-β blockade was used to demonstrate a reduction in the generation of T_RM_ populations, in conjunction with significantly decreased tumor protection. Finally, parabiosis was used to demonstrate that vaccinated mice were protected against tumor rechallenge, while no protection was afforded to parabiosed naive mice. These studies thus convincingly showed a dominant role for E7-specific T_RM_ cells in protection against orthotopic head and neck cancer.

A thorough investigation of VACV-OVA vaccination route separately implicated T_RM_ cells as important players in anti-tumor immunity (110). Mice infected with VACV-OVA by the dermal, nasal, or peritoneal routes showed distinct patterns of antigen-specific T cell memory formation in circulation and in peripheral tissues (110). Using i.p. vaccination to generate circulating memory without resident memory, or FTY720 as a means for blocking T cell access to the skin, it was shown that either circulating or resident memory are sufficient for protection against B16-OVA re-challenge in the skin (110). Moreover, parabiotic transfer of circulating memory to naïve recipient mice conferred reduced tumor protection compared with vaccinated parabiotic donor mice, demonstrating that T_RM_ cells are significant contributors to tumor immunity induced by viral vaccination (110).

**Figure 2:**
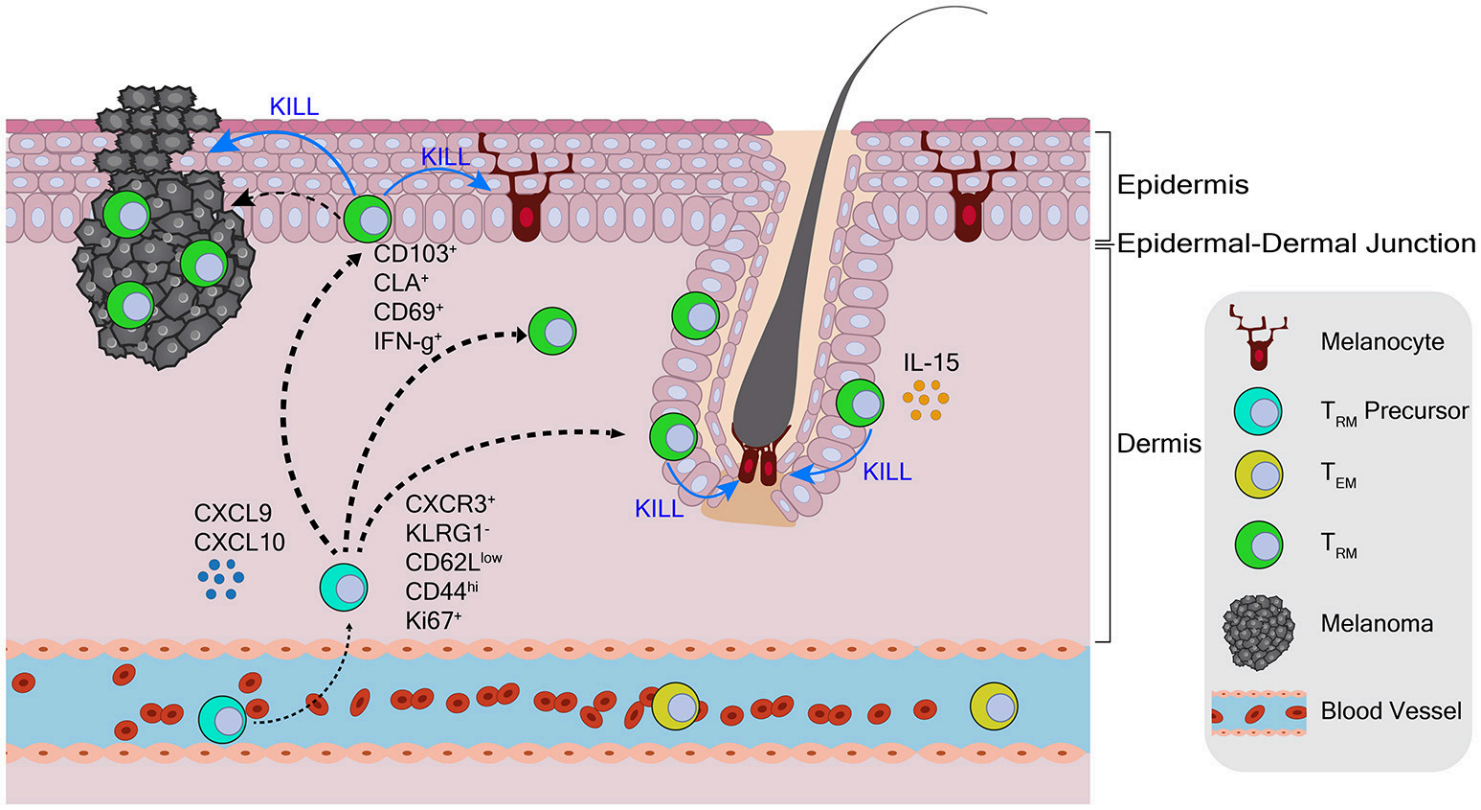
Skin resident-memory T cell responses to melanoma in the context of autoimmune vitiligo. Tumor-specific T_RM_ precursor populations are recruited from circulation into the skin by chemokine signals. Following entrance into the skin, these T cells kill melanocytes, develop into T_RM_ cells, and produce IFN-γ. Tumor-specific T_RM_ cells populate the dermis, epidermis, and hair follicles, where IL-15 is produced. CD103-dependent T_RM_ cells mediate the durable recall response to melanoma in the dermis.

This same year, our own work illustrated that T_RM_ cells in the skin are both necessary and sufficient for long-lived protection against B16 melanoma (111). Employing therapeutic depletion of regulatory T cells to break tolerance to melanoma differentiation antigens, followed by surgery to curatively excise residual B16 primary tumors, we identified the formation of tumor/self (gp100) Ag-specific CD8 T cells in the skin with a CD44^hi^ CD62L^low^ CD103^+^ CD69^+^ CLA^+^ T_RM_ phenotype (Figure 2) (111). These tumor-specific T cells persisted for several months even following extended FTY720 treatment or upon skin grafting onto T cell deficient mice, indicating their true T_RM_ nature. Importantly, generation of this T_RM_ population in the skin depended on CD8 T cell expression of CD103 and Fut7 (the enzymatic determinant of cellular CLA production) (111). As Treg-depleted mice also generated memory T cell responses in lymphoid tissues, a requirement for T_RM_ cells in protection against B16 dermal re-challenge was shown by two methods. First, long-lived tumor immunity was shown to be unperturbed by the continual administration of FTY720. Second, genetic knockout of CD103 in the mouse CD8 T cell compartment, while having no effect on lymphoid memory generation, was shown to completely abrogate skin tumor protection. Thus, CD103-dependent T_RM_ cells in the skin were the sole mediators of long-lived immunity against the dermal B16 melanoma (111).

This study also extended a link between tumor immunity and autoimmunity formed by our earlier work that autoimmunity against normal melanocytes (i.e., vitiligo) maintains lymphoid memory T cell responses against melanoma/melanocyte shared antigens (112, 113). Indeed we found that T_RM_ cells only developed in the skin of mice with treatment-related vitiligo (111). Vitiligo was shown to be required for the seeding of gp100-specific T_RM_ precursors throughout pigmented and depigment skin, but preferentially in melanocyte-depleted hair follicles (111) (Figure 2). The concept of generating T_RM_ responses against tumor/self-antigens was also illustrated in studies involving intradermal DNA vaccination against gp100 (114). This study showed that vaccination induced the development of gp100-specific T_RM_ cells in the skin, in association with autoimmune vitiligo. Importantly T_RM_ cells (but not circulating memory T cells) were refractory to low dose anti-CD8 mAb depletion, a technique used to demonstrate that T_RM_ cells mediate long-lived protection against B16 tumor rechallenge (114). Thus, in the generation of protective T_RM_ responses to tumor antigens that are shared by normal tissues, autoimmunity clearly plays an important role.

These mechanistic studies in mouse tumor models, coupled with data from patient tumor TILs, now clearly affirm the relevance of T_RM_ cells to tumor immunity. Preclinical immunotherapy studies further illustrate that established methods for vaccinating against tumor antigens can be highly effective at generating T_RM_ responses to cancer (93, 110, 114). Reaffirming studies of 15 years ago, vaccination route is crucial for generating the proper T_RM_ responses to tumors in various tissue locations (106). Treg depletion also generates T_RM_ responses against shared tumor/self-antigens, likely relating to the role of Tregs in controlling peripheral tissue autoimmunity (111). While correlative data support the notion that immune checkpoint inhibitor therapies act on phenotypically exhausted T_RM_-like TILs in solid tumors, such therapies have not yet been shown to induce *de novo* T_RM_ responses in cancer patients. Regardless, the knowledge that ~20% of anti-PD-1 treated melanoma patients develop vitiligo (115) may imply that melanoma-specific T_RM_ responses are generated or awakened in such patients. Indeed, skin immune-related adverse events of ICI therapy have excellent prognostic value for melanoma patients (116), further underscoring the idea that autoimmunity supports T_RM_ responses to cancer. The next section will discuss a role for T_RM_ cells in mediating autoimmunity; a class of diseases that has long instructed the field of tumor immunology.

## Role of T_RM_ cells in autoimmune disease

### Skin autoimmunity

Skin autoimmune diseases provide the most compelling evidence of T_RM_-mediated pathogenic responses against self. In accordance with the above studies in melanoma, three recent studies have demonstrated melanocyte Ag-specific CD8 T_RM_ cells in vitiligo-affected patient skin (non-melanoma associated) (117–119). The first of these showed that vitiligo-associated T_RM_ cells display a CD8^+^CD103^+^CD49a^+^ phenotype, and become localized to both the epidermis and dermis of lesional patient skin (117). These cells are highly functional based on their production of perforin, granzyme B, and IFN-γ upon *in vitro* restimulation (117). T_RM_ cells in vitiligo-affected skin also expressed the tissue homing receptor CXCR3 (118), consistent with prior reports that vitiligo is mediated by IFN-γ induced ligands for this receptor; CXCL9 and 10 (120). Additional phenotypic analysis of vitiligo-associated T_RM_ cells revealed the expression of CD122, the alpha chain of IL-15R (119); a classic memory T cell marker that supports T_RM_ populations in viral infection models (20, 46). Importantly, mechanistic studies in a T cell receptor transgenic (TCR Tg) CD8 T cell-induced mouse model of vitiligo, revealed that anti-CD122 mAb treatment repigmented vitiligo-affected skin in a highly durable fashion (119). This study was the first to show that factors supporting T_RM_ cell maintenance can serve as targets to impair autoimmunity. Interestingly no differences were observed between T_RM_ responses in patients with active vs. stable disease (118, 119), further underscoring the concept that vitiligo is a disease of immune memory.

Corollary studies of CD8 T cell responses in psoriasis patients show that not all T_RM_ responses are created equal. Psoriasis, like vitiligo, occurs in lesional patches of skin, but is recognized as an IL-17-driven disease. Accordingly CD8^+^ T_RM_ cells in psoriatic plaque skin preferentially produced IL-17 upon restimulation, exhibit a CD103^+^ CD49a^−^ CLA^+^ CCR6^+^ IL-23R^+^ phenotype (117, 121), and lack expression of CXCR3 (118). Interestingly, cytokine production was maintained by psoriasis-associated T_RM_ cells even in patients that had undergone long term treatment and had resolved disease (117, 118), again supporting the highly durable nature of T_RM_ responses in autoimmune disease. Contrary to the long-held belief that psoriasis is mediated by γδ T cells, active and clinically resolved psoriasis lesions were shown to contain oligoclonal T cell populations that were overwhelmingly of the αβ T cell lineage, and that produced both IL-17 and IL-22 (122). These studies collectively indicate a role for Tc17-like T_RM_ cells in the pathogenesis of psoriasis.

Although less well-studied, T_RM_ responses have recently been reported in conjunction with scleroderma—a disease of unclear etiology, characterized primarily by fibrosis of the skin. Analysis of skin from patients with early stage scleroderma revealed higher proportions of CD8^+^CD28^−^ T cells that expressed the T_RM_ marker CD69, and also overwhelmingly expressed CCR10, although largely lacked CD103 (123). Functional analyses indicated a proportion of restimulated cells that were capable of making granzyme B, IFN-γ and IL-13, a pro-fibrotic cytokine (123). This study, together with the above studies in vitiligo and psoriasis patients, highlights the diverse functional programs adapted by T_RM_ cells in the context of aberrant skin pathologies.

### Other autoimmune and inflammatory conditions

Several reports have provided evidence of T_RM_ responses in other autoimmune diseases and inflammatory conditions, particularly in diseases of the brain and CNS. With growing evidence that CD8 T cells may play a role in the pathogenesis of multiple sclerosis (124), work in mouse MS models has suggested that Ag-specific CD8 T_RM_ cells in the CNS could contribute to pathogenesis (125). The creation of TCR Tg mice expressing a TCR specific for an MHC-I restricted epitope of an astrocyte-expressed protein resulted in MS-like disease, with the brains of mice becoming infiltrated by CD8 T cells with a CD44^+^CD69^+^CD103^+^ T_RM_ -like phenotype (125). Evidence of MS-associated T_RM_ cells also derives from lesional brain specimens of MS patients. In a small subset of patients with high inflammatory infiltrates, CD103-expressing CD8 T cells were identified, although these cells did not express CD69 (126). In addition to MS, a study of Rasmussen's Encephalitis, a rare pediatric neuroinflammatory disease of unknown etiology, also reported CD8 αβ T cells in seven out of seven RE brain surgery specimens, >50% of which expressed CD103 (127). Mouse studies show that T_RM_ cells may accumulate naturally with age in the CNS, evidenced by the appearance of a CD8 ^+^CD44^hi^ CD62L^low^ CD69^hi^ PD1^+^ subset (128). In an ischemic stroke model in aged mice, restimulation of brain CD8 T cells induced the production of TNF-α, IFN-γ, and CCL2 (128). Despite this, the question remains of whether brain CD8 T_RM_ cells are pathogenic. CD8^+^CD103^+^ T_RM_ cells from brains of mice with MS-like disease did not produce cytokines (125). In lupus-prone mice, CD8^+^CD44^hi^CD62^low^CD69^+^ T_RM_-like cells accumulated in the brain, but their ablation exacerbated neuropsychiatric lupus, suggesting that these cells might instead serve a regulatory role (129).

As of yet, few studies have convincingly identified T_RM_ cells in other autoimmune disease types. An investigation of lesional biopsies from recent-onset type-1 diabetes (T1D) patients reported CD8^+^CD69^+^CD103^+^ T_RM_-like cells in diseased islets (130), although a separate study showed a preponderance of CD8 T cells with similar phenotypes in normal, healthy human islets (131). Transcriptomes of T1D patient islets were more skewed to the production of inflammatory cytokines including IFN-γ IL-18, and IL-22 (130), although it is as yet unclear if these pathogenic cytokines are derived from T_RM_ cells. It has been speculated that T_RM_ cells mediate, or contribute to, a host of human autoimmune and inflammatory conditions (132). Advancements in our understanding of T_RM_ biology should guide further investigations of T_RM_-mediated pathology.

T_RM_ responses against normal self-tissues can be instructive with regards to cancer immunity. First, they reveal that self Ag-specific T cells can be maintained as T_RM_ responses, in the peripheral tissues where carcinomas arise (Figure 1). Such T cells would seem ideally situated to provide surveillance against tumorigenesis, progression, and metastasis, and may naturally do so. Second, they illustrate that T_RM_ cells can adopt a number of stable immune effector states, producing IFN-γ, IL-17, IL-22, IL-13, and/or granzyme B, all of which have separately been shown to oppose tumor growth. In particular, vitiligo and psoriasis-associated T_RM_ cells maintain stable phenotypes and provide durable recall responses, with more apparent functional diversity than was originally recognized based on viral infection models. Our knowledge of mechanisms driving pathogenic self Ag-specific T_RM_ cells, coupled with our understanding of T_RM_ responses against foreign infections, can greatly inform our understanding of T_RM_ responses to cancer.

## Optimizing T_RM_ cell responses for cancer immunotherapy

The discovery that standard cancer vaccine and immunotherapy approaches provide long-lived protection through T_RM_ responses—rather than T_CM_ responses, as originally speculated—indicates that promoting T_RM_ responses is a worthwhile goal for the field. The challenge remains in generating T_RM_ responses when (and where) they don't naturally develop. The final section of this review will highlight current knowledge regarding T_RM_ cell behavior that can aid in promoting such responses in the setting of cancer immunotherapy.

### Optimal T_RM_ cell precursor populations

The ultimate goal of adoptive T cell therapy is to provide long-lived cancer cures. In patients with metastatic solid tumors, T cells must seek and destroy tumor cells in multiple peripheral tissue locations. T_RM_ cells seem ideal for this job, although successfully delivering these populations presents clear challenges. Because T_RM_ cells are, by definition, resident in peripheral tissue, their *in vitro* generation for intravenous delivery is a complex proposition. In considering the systemic administration of a T_RM_ response, one must consider the precursor populations that can optimally seed tissues in a receptive host.

#### T_RM_ precursor seeding

Studies in mouse models largely support the conclusion that timing is crucial for T_RM_ precursor seeding, and that this event must occur early during the course of T cell priming and differentiation. In LCMV infection, early effector cells migrated into intestinal epithelium, seeding a T_RM_ population within 7 days of the initial infection (41). Similarly, in HSV infection, a delay of 2 weeks did not alter T_CM_ formation, but it dramatically reduced T_RM_ cell seeding in the skin, resulting in a loss of protection against viral rechallenge (32). This window of opportunity is affirmed by studies of mucosal chlamydia vaccination, wherein T_RM_ precursors seeded uterine mucosa within 7 days of vaccination (24). Our own studies indicate that tumor-specific T_RM_ precursors are primed early in response to immunotherapy, as they could be isolated from melanoma-draining lymph nodes just 8 days following therapeutic depletion of regulatory T cells (111). These studies support the idea that T cells at an early differentiated state have the greatest propensity to form T_RM_.

Despite this, some evidence supports the idea that T_CM_ cell plasticity can give rise to T_RM_. In studies employing VACC-OVA as a dermal vaccine, sorted OVA-specific T_CM_ cells that were re-transferred *in vivo*, generated skin T_RM_ populations in response to VACC-OVA recall infection (110). On a per-cell basis, T_CM_ cells were shown to be more effective at generating T_RM_ cells compared with naïve T cells (110). Similarly, when administered to tumor-bearing mice, Ag-specific T_CM_ cells were better at accessing tumors and acquiring a T_RM_ phenotype, compared with their naïve counterparts. As T_RM_ and T_CM_ cells represent distinct lineages, it remains unclear how such memory T cell reprogramming occurs. While it has been shown that T_RM_ and T_CM_ cells have a common clonal origin (133), T_CM_ cells do not naturally convert to T_RM_ cells in the post-infection setting (17). Thus, factors relating to secondary infection with VACC-OVA, or tumor growth itself, may alter the plasticity of T_CM_ cells (110). Indeed prior preclinical approaches to melanoma adoptive immunotherapy with *in vitro* generated gp100-specific T_CM_, T_SCM_, and Tc17 subsets (12, 15, 134) may naturally give rise to T_RM_ cells in a tumor-bearing host. This is consistent with the observation that these treatments all induce overt vitiligo (12, 15, 134)—a disease of skin-resident memory.

#### T_RM_ precursor phenotypes

With regards to phenotype, epithelium-infiltrating T_RM_ precursors in infectious disease models have been shown to express CXCR3 and lack expression of the terminal effector T cell marker KLRG1 (20, 111). Deficiency in CXCR3 reduces the overall number of T_RM_ cells in the skin (20, 135, 136), and mAb-mediated CXCR3 blockade can prevent T_RM_ formation (34). Accordingly, we identified CXCR3^+^KLRG1^−^ T cells in tumor-draining lymph nodes, that were capable of seeding tumor-specific Trm responses in the skin following i.v. adoptive transfer (Figure 2) (111). CXCR3 is also reported as a mediator of T cell access to solid tumors (137), thus its role in the seeding of T_RM_ precursors further underscores the importance of CXCR3 expression on T cells in an immunotherapy setting.

In our melanoma model, we also observed CD103 expression on T_RM_ precursors in tumor-draining lymph nodes, and found that genetic loss of CD103 impaired early T cell lodgement in the skin (111). This is in contrast to viral models in which CD103 is only expressed upon T cell entry into the epidermis (20), although we also observed a further increase in CD103 expression following T cell entry into the skin. As our T_RM_ cells were primed in response to dermal melanoma growth, early CD103 expression in our model could result from tumor-derived TGF-β entering tumor-draining lymph nodes. Numerous infectious disease studies have shown that CD103 expression is required for the long-lived, antigen-independent maintenance and retention of T_RM_ cells (20, 47, 101, 138, 139). However, this unexpected role for CD103 in promoting skin lodgment indicates that CD103 may also be a useful feature of tumor-specific T_RM_ precursors.

#### T_RM_ precursor transcriptional programs

In considering transcription factors that program T_RM_ development, whereas *Hobit* expression is restricted to late events in peripheral tissues (55), *Runx3* expression occurs earlier, and appears to program T_RM_ precursor behavior. Identified by an siRNA screen of factors that promote tissue residence *in vivo*, Runx3 was found to suppress *Tbet* expression, enforce *Itgae* expression, and suppress multiple genes associated with tissue egress (54). Runx3 expressing T cells were transcriptionally distinct from T_CM_ and T_EM_ precursor populations as early as 7 days post infection. Of therapeutic importance, Runx3 overexpression in LCMV-specific CD8 T cells promoted T cell access to viral antigen-expressing B16 tumors, enforced the acquisition of a T_RM_ phenotype in tumors, and enhanced T cell anti-tumor activity (54). These studies establish that Runx3 promotes T_RM_ characteristics and anti-tumor efficacy of transferred T cells (54), making it an attractive target for expression in the adoptive immunotherapy setting.

### Optimal host tissue microenvironment

Tumor cell dissemination from primary tumors to peripheral tissue locations is predicated on Paget's seed and soil hypothesis of 1898, which states that tumor cells (the seed) can only take up residence in suitable tissues (the soil) (140). This principle can also be applied to T_RM_ cell seeding in peripheral tissues. Indeed among T cells, T_RM_ cells are unique in their propensity for tissue. Providing the proper “soil” for precursor T cell residence will be a critical step toward supporting T_RM_ function, both in tumors and in cancer-prone tissues. Moreover, the properties of T_RM_ cells that enable them to function in diverse peripheral tissue niches, might imbue them with the unique ability to persist and function in solid tumors.

#### Metabolic factors

Metabolic characteristics of T_RM_ cells that support their function in peripheral tissues may also support their function in tumors. In a VACV skin infection model, fatty acid binding protein 4 and 5 (*FABP4/5*) were shown to be among the highest expressed genes in T_RM_ cells, enabling the metabolism of exogenous free fatty acids in the skin (51). T_RM_ cells in the tumor microenvironment must compete for nutrients with tumor cells, which use high levels of glucose and glutamine (141). Indeed Tregs have been shown to function well in the TME due to their ability to utilize both glycolysis and fatty acid metabolism (142). Reliance on fatty acid catabolism has recently been shown to be essential for CD8^+^ TIL function (143). The PPAR agonists fenofibrate, and bezafibrate, which both promote fatty acid oxidation, have each been shown to improve T cell anti-tumor activity (143, 144), although it remains to be seen if these effects are due to improved T_RM_ responses. Regardless, the metabolic requirements of T_RM_ cells may make them ideally suited to persist and function in a metabolically hostile TME.

#### Chemokines and cytokines

Chemokine cues are crucial for the seeding of T_RM_ precursors, and can be used therapeutically to pull T_RM_ cells into peripheral tissues. Our finding that melanoma-specific CXCR3^+^ precursors cells only induce T_RM_ responses in vitiligo-affected hosts (111), together with studies showing that the CXCR3/CXCL9/10 axis is crucial for vitiligo development (120, 145), underscores how an autoimmune tissue microenvironment can provide a hospitable niche for tumor-specific T_RM_ cell seeding (Figure 2). CXCL9 and 10 have also been used therapeutically to seed T_RM_ responses based on a “prime and pull” approach (146). In one study, subcutaneous vaccination elicited a systemic T-cell response against HSV, followed by topical CXCL9/10 application to the vaginal mucosa as a means to pull activated T cells into tissue (146). The resulting T_RM_ responses and long-term protection against HSV were comparable to that of mice that that had been immunized intravaginally (146). Similarly, when immunizing against mTB in the lung, parental vaccination followed by intranasal administration of CXCL16, pulled CXCR6 cognate receptor-expressing T_RM_ cells into the lung where they provided long-lived protection (147). CXCR10 has separately been shown to promote T_RM_ formation in the skin (135, 148), suggesting the possibility of a similar approach involving CCL27. These studies show that, even when precursors are generated in a systemic manner, chemokine signals in a specific tissue location can induce functional T_RM_.

Inflammatory sensitizing agents can also modify peripheral tissue in a way that promotes robust T_RM_ responses. Using the chemical sensitizing agent dinitrofluorobenzene (DNFB), local inflammation in skin recruited effector T cells and converted them to a T_RM_ phenotype (32). Similarly, topical diphenyl cyclopropinone (DPCP) applied to human skin induced contact dermatitis and the subsequent formation of T_RM_ (133). Accordingly, DPCP application to human skin has been shown to induce high levels of cutaneous CXCL9 and 10 (149). Interestingly, DPCP recently received orphan drug approval for the topical treatment of cutaneous melanoma metastases (150), having demonstrating dramatic local efficacy against cutaneous melanoma metastases in one patient that received concurrent immune checkpoint inhibitor therapy (151). It is tempting to speculate that T_RM_ cells participated in this anti-tumor response, which was also accompanied by vitiligo (151).

Once T cells access peripheral tissues, both TGF-β and IL-15 are recognized as fundamentally important T_RM_ survival cues (20, 56). While TGF-β has come to be known for its roles in promoting tumor growth (152), suppressing T cell function (153), and enforcing Treg stability (154), its key role in T_RM_ cell generation suggests its value in certain immunotherapy contexts. On the other hand, IL-15 has long been recognized for its role in supporting anti-tumor T cell responses (155, 156). Studies show that T_RM_ cells preferentially accumulate at sites of high IL-15 production, such as hair follicles in the skin (46, 111). Accordingly, we found that melanoma/melanocyte (gp100)-specific T_RM_ cells cluster in hair follicles of vitiligo-affected skin (111). In conjunction with the finding that gp100-specific T cells express CD122 (IL-15Ra) and require IL-15 for their pathogenic role in melanocyte destruction (119), IL-15 may also enhance melanoma-specific T_RM_ maintenance. Although it is important to note exceptions to the requirement for IL-15, which are surprising considering its canonical role as a homeostatic memory cytokine (157). In fact, certain T_RM_ responses (i.e., in the female reproductive tract) exhibit no dependence on IL-15 for proliferation or survival (157). T_RM_ cells that re-engage antigen in peripheral tissues appear to decrease their reliance on IL-15 (157), indicating that antigen also serves an important role in peripheral tissue.

#### Antigen and costimulatory requirements

As T_RM_ precursors seed tissue, their differentiation and maintenance has been shown to be shaped by the local engagement of Ag. Indeed following VACV infection, T_RM_ responses were increased ten to 50-fold if their cognate antigen had been engaged in the skin (158, 159). We also observed an important role for peripheral antigen in generating tumor-specific T_RM_ responses (111). Following priming in response to a B16-OVA tumor in the dermis, T_RM_ cells that re-engaged their target antigen on melanocytes in the skin (gp100-specific) were present in far greater numbers than those that could not (OVA-specific) (111). In vaccinating against a tumor-specific neoantigen, T_RM_ responses in peripheral (non-tumor) tissues might best be generated by a proposed “prime and trap” approach (34). This was demonstrated in the context of malaria vaccination, wherein the expression of cognate antigen on hepatocytes served to trap circulating CD8 effector T cells in the liver, where they underwent conversion to T_RM_ cells (34).

While memory T cells are defined by their ability to persist in the absence of Ag, in some settings chronic Ag exposure might also support T_RM_ cell persistence. Following HPV vaccination of patients with cervical intraepithelial neoplasia, CD8^+^ T cells expressed CD69, and localized to tertiary lymphoid structures in neoplastic cervical tissue, where they expressed Ki67 as evidence of cognate antigen engagement (160). In considering T_RM_ responses against a tumor/self Ag, we showed that functional T_RM_ could be generated against a melanoma antigen (gp100), but only in conjunction with autoimmunity against normal host melanocytes (111). While this may reflect the chemokine/cytokine environment of the autoimmune tissue niche, it may alternately reflect a role for persistent antigen exposure. Interestingly, we identified melanoma-specific T_RM_ cells throughout the skin, although they were preferentially localized to hair follicles containing white hairs, suggesting an absence of local antigen (111). It remains possible that, in the context of autoimmune disease, T_RM_ cells develop into both Ag-dependent and independent subsets.

Although less well-studied, peripheral costimulation may also play a crucial role in programming the T_RM_ response. Recent studies of intranasal flu vaccination showed that boosting with 4-1BBL in a replication defective adenovirus vector generates a robust lung parenchymal CD69^+^ CD103^+/−^ T_RM_ population (161). Boosting required local (intranasal) 4-1BBL installation, and worked by recruiting additional Ag-specific T cells from circulation into the lungs (161). Lung T_RM_ responses generated in this fashion were highly durable and provided protection for at least 1 year after boost (161). Earlier work also suggests that agonistic OX-40 mAb treatment can promote lung T_RM_ responses, although these studies involved parenteral vaccination (162). Future work is needed to determine how and where to provide the most potent costimulatory signals for optimal T_RM_ formation.

## Conclusions

The above findings represent a fundamental advance in our understanding CD8 T cell responses to cancer. While the success of ICI therapy has reaffirmed the long-held belief that CD8 T cells are crucial for tumor immunity, an evolving knowledge of memory T cell function in peripheral tissues has informed our understanding of the type of T cell that may be most needed. Innovations in single cell cytometry and RNA sequencing have rapidly been brought to bear on the characterization of tumor-infiltrating lymphocytes and, together with mechanistic studies in mice, provide compelling evidence that T_RM_ cells are players in the immune response to human cancer.

Several key questions remain. While T_RM_-like TILs portend improved prognosis for a growing number of cancers, future studies should address how to fully revive such cells in the TME, and how to generate greater numbers of T_RM_ precursors through immunotherapeutic means. A role for T_RM_ cells in tumor immunosurveillance has not yet been established, and it will be interesting to learn if CD8 T_RM_ cells can also limit tumorigenesis. Finally, the field has focused heavily on CD8^+^ T cells, although a role for CD4 T_RM_ populations is not yet understood. Local T_RM_ helper subsets might be greatly beneficial to tumor immunity, whereas T_RM_ regulatory cells might be particularly detrimental in the TME.

The knowledge that T_RM_ cells can be generated by cancer vaccine and immunotherapy regimens represents a paradigm-shift for a field that has long monitored tumor-specific T cells in the blood. Thus, going forward one must recognize a need to monitor T_RM_ responses in peripheral tissues and tumors of cancer patients receiving immunotherapy. Such peripheral T cell responses might provide the best indication of responsiveness to therapy, and long-term survival. Informed by studies in infectious disease models, and instructed by the involvement of T_RM_ cells in autoimmunity, future research efforts will hopefully overcome the barriers to promoting effective T_RM_ responses to cancer.

## Author contributions

AM and MT both conceptualized, wrote, revised, and approved of the final submitted manuscript.

### Conflict of interest statement

The authors declare that the research was conducted in the absence of any commercial or financial relationships that could be construed as a potential conflict of interest.
